# Medial septum glutamatergic neurons modulate nociception in chronic neuropathic pain via projections to lateral hypothalamus

**DOI:** 10.3389/fphar.2023.1171665

**Published:** 2023-05-17

**Authors:** Bing-Qian Fan, Jun-Ming Xia, Dan-Dan Chen, Li-Li Feng, Jia-Hui Ding, Shuang-Shuang Li, Wen-Xian Li, Yuan Han

**Affiliations:** Department of Anesthesiology, The Eye and ENT Hospital of Fudan University, Shanghai, China

**Keywords:** glutamatergic, lateral hypothalamus, medial septum, neuropathic pain, hyperalgesia, supramammillary nucleus

## Abstract

The medial septum (MS) contributes in pain processing and regulation, especially concerning persistent nociception. However, the role of MS glutamatergic neurons in pain and the underlying neural circuit mechanisms in pain remain poorly understood. In this study, chronic constrictive injury of the sciatic nerve (CCI) surgery was performed to induce thermal and mechanical hyperalgesia in mice. The chemogenetic activation of MS glutamatergic neurons decreased pain thresholds in naïve mice. In contrast, inhibition or ablation of these neurons has improved nociception thresholds in naïve mice and relieved thermal and mechanical hyperalgesia in CCI mice. Anterograde viral tracing revealed that MS glutamatergic neurons had projections to the lateral hypothalamus (LH) and supramammillary nucleus (SuM). We further demonstrated that MS glutamatergic neurons regulate pain thresholds by projecting to LH but not SuM, because the inhibition of MS-LH glutamatergic projections suppressed pain thresholds in CCI and naïve mice, yet, optogenetic activation or inhibition of MS-SuM glutamatergic projections had no effect on pain thresholds in naïve mice. In conclusion, our results reveal that MS glutamatergic neurons play a significant role in regulating pain perception and decipher that MS glutamatergic neurons modulate nociception via projections to LH.

## Introduction

As a leading cause of years lost to disability, chronic pain imposes a massive personal and economic burden on more than 30% of people worldwide (Murray et al., 2013; Cohen et al., 2021). Neuropathic pain is chronic pain caused by primary lesions or nervous system dysfunction associated with sensory abnormalities (Baron, 2006; Lu et al., 2017; Cohen et al., 2021). Although multiple pharmacological and non-pharmacological therapies proposed a solution for reducing neuropathic pain, treating this condition remains challenging for physicians cause a sufficient number of patients failed to experience satisfactory pain relief after treatment (Baron et al., 2010; Culp et al., 2020; Fitzcharles et al., 2021; Jiang et al., 2022). Thus, exploring the neural mechanism of neuropathic pain may lead to discovering novel pharmacological therapeutic targets.

The medial septum (MS), mainly composed of cholinergic, glutamatergic, and GABAergic neurons, is implicated in a variety of functions such as sensorimotor integration, affect-motivation and cognition (Freund, 1989; Kiss et al., 1990; Dutar et al., 1995; Colom et al., 2005; Hasselmo, 2006; McNaughton et al., 2006; Calandreau et al., 2007; Braida et al., 2014; Ang et al., 2017; Danisman et al., 2022). Studies of deep brain stimulation have shown that medial septal stimulation immediately relieves chronic pain in patients (Gol, 1967; Schvarcz, 1993). Besides, MS lesion attenuated formalin-induced theta activation and pyramidal cell suppression in dorsal hippocampus field CA1, while muscimol or zolpidem microinjection into MS suppressed formalin-induced nociceptive behavior (Zheng & Khanna, 2001; Lee et al., 2011). Moreover, another study reported that muscimol microinjection into the MS reversed peripheral hypersensitivity evoked by chronic constriction injury (CCI) (Ariffin et al., 2018). These findings indicate a potential link between MS and chronic neuropathic pain providing a critical neurophysiologic basis of chronic neuropathic pain responsible for the manifestations of neuronal plasticity changes (Xia et al., 2016). Ionotropic glutamate receptors, especially N-methyl-D-aspartate receptors expressed on postsynaptic membranes and related to neuronal plasticity, are involved in pain-related processes (Hansen et al., 2014; Xia et al., 2016; Li et al., 2019). Furthermore, nonselective and selective N-methyl-D-aspartate receptor antagonists have been shown to mitigate neuropathic pain (Collins et al., 2010; Rondon et al., 2010). In line with all the evidence, we hypothesize that MS glutamatergic neurons might be involved in regulating neuropathic pain sensitization.

The current study aimed to investigate the cellular level mechanisms of MS glutamatergic neurons in regulating neuropathic pain sensitization. First, using c-Fos staining, we identified the activity changes of MS glutamatergic neurons in CCI animals. Moreover, chemogenetic manipulations demonstrated that MS glutamatergic neurons contribute in regulating pain sensitization of naïve and CCI animals. Hence, optogenetic investigations revealed that MS glutamatergic neurons regulate pain sensitization by projecting to the LH but not SuM. All this evidence was sufficient to provide and demonstrate a novel neuronal and neural circuit mechanism that modulates the perception of chronic neuropathic pain.

## Materials and methods

### Animals

The Vglut2-Cre mice (Vglut2, vesicular glutamate transporter 2; #028863) were obtained from Jackson Laboratory. Adult male mice (2–6 months old) were group-housed (≤ 5 per cage) on a 12 h light/dark cycle with food and water available *ad libitum*. These mice were randomly assigned to the nominated groups described in the following experiments. All procedures were performed under international guidelines on the ethical use of animals and approved by the Animal Care and Use Committee of Fudan University (Approval No. SYXK20200032). Efforts were taken to minimize animal suffering and the testing procedure adheres to principles of animal ethics and the 3Rs.

### Chronic constriction injury (CCI) model

To establish a neuropathic pain model, CCI surgery (Chronic constriction injury of the sciatic nerve) was performed as described previously (Bennett and Xie, 1988; Wang et al., 2021a). In brief, mice were anesthetized with sodium pentobarbital (50 mg kg^−1^ i.p.). The left sciatic nerve was exposed at the mid-thigh level by blunt dissection. Three nonabsorbable 4–0 silks were loosely tied around the sciatic nerve at ∼1.0 mm intervals. Sham procedures (sciatic nerve exposure without ligation) were performed as controls. After suturing, erythromycin ointment was applied locally to keep the wound from infection. Finally, mice were placed in a clean and warm cage to recover from the anesthesia.

### AAV vectors

In this study, adenovirus-associated virus (AAV) vectors were utilized, including AAV2/9-EF1α-DIO-EGFP (PT-0795; Brain VTA, China), AAV2/9-EF1α-DIO-mCherry (PT-0013; Brain VTA, China), AAV2/9-EF1α-DIO-hM3Dq-mCherry (PT-0042; Brain VTA, China), AAV2/9-EF1α-DIO-hM4Di-mCherry (PT-0043; Brain VTA, China), AAV2/9-flex-taCasp3-TEVp (PT-0206; Brain VTA, China), AAV2/9-hSyn-DIO-mGFP-T2A-Synaptophysin-mRuby (PT-1244; Brain VTA, China), AAV2/9-EF1α-DIO-hChR2(H134R)-mCherry (PT-0002; Brain VTA, China), and AAV2/9-EF1α-DIO-NpHR3.0-mCherry (PT-0007; Brain VTA, China). The titer of all AAV vectors ranged from 1 to 5 × 10^12 genomic copies per milliliter.

### Stereotaxic surgery and microinjection

Male mice (2–3 months old, 22–28 g) were anesthetized and stabilized in a stereotaxic frame (RWD Life Technology Co., Ltd., Shenzhen, China). The eyes were protected with erythromycin ointment. After exposing the skull’s cranium, 3% hydrogen peroxide was applied to remove the periosteum, and the residual was washed off by normal saline. For the microinjection, the AAV vectors (∼100 nL) were injected into MS (AP = +0.88 mm; ML = +0.55 mm; DV = −3.7 mm, 8° angle) at a rate of 1 nL sec-1 via a glass pipette connected to a programmable auto-nanoliter Injector (Nanoject III, Drummond, United States), followed by a 10-min pause to minimize backflow. The optic fibers were implanted above the LH (AP = −1.35 mm; ML = ±1.05 mm; DV = −5.10 mm) or SuM (AP = −2.70 mm; ML = +0.25 mm; DV = −4.50 mm) through dental cement. Mice were kept in their home cages after fully awake. After experiments, histological analysis was performed to verify the locations of viral transduction and optical fibers. Data were excluded for analysis if viral transduction extended beyond the MS brain regions or the locations of optical fibers were out of LH or SuM.

### Chemogenetic manipulation

After mCherry, hM3Dq-mCherry or hM4Di-mCherry expression in MS for 3 weeks, saline or clozapine *N*-oxide (CNO) (Brain VTA, China) 1 mg kg^-1^ were intraperitoneally injected at least 30 min (Tang et al., 2021) before behavioral tests. Experimenters were blind to saline or CNO administered during behavioral tests.

### Optogenetic stimulation

Optical fibers in LH or SuM were connected to a blue (473 nm) or yellow (589 nm) laser generator (Newdoon, Hangzhou, China) through optical cables (Aoguan, Nanjing, China). A blue laser with 5 ms width (473 nm, 3–5 mW, 10 Hz) was synchronized for optogenetic activation during behavioral measurements. For optogenetic inhibition, a constant yellow laser (589 nm, 5–7 mW, 8s-on/2s-off) was applied synchronized during behavioral measures.

### Behavioral tests

#### 50% Paw withdraw threshold (50% PWT)

A simplified up-down method with von Frey filaments was used to estimate the mechanical hyperalgesia of mice. Briefly, mice were placed in polyethylene cages separately on an elevated metallic wire mesh platform in a quiet environment. Before testing, mice were allowed to acclimatize to the environment for 1–2 h. The test started with the midrange filament of 0.16 g strength. Subsequent filaments were proceeded according to the up-down method, and 5 consecutive touches were applied at 5 min intervals for rest. The filaments were pressed against the plantar surface and held for 3 s. Positive responses were noted when mice withdrew their hind paws during this time. Finally, 50% PWTs were calculated as described previously (Bonin et al., 2014). Behavioral tests were carried out in a blinded manner. Detailed timelines for each experiment are presented in Figures.

#### Paw withdrawal latency

To assess thermal nociception, paw withdrawal latencies (PWLs) were measured using the Hargreaves test (Hargreaves et al., 1988) with an IITC plantar analgesia meter (IITC Life Science). Room temperature was controlled at 23°C ± 2°C. Mice were individually placed in polyethylene cages on a glass platform and allowed to accommodate the apparatus for 1–2 h. A radiant heat source beneath the glass was used to stimulate the plantar surface of the hind paw. In advance, heat intensity was adjusted to produce a baseline of 10–15 s. To prevent tissue damage, the cutoff time was set to 20 s. Flinching, flicking, and trembling were considered as positive responses. The measurements were triplicated at 10 min intervals, and the mean was calculated as the PWL.

### Immunohistology and confocal imaging

The mice were deeply anesthetized using pentobarbital sodium (60 mg kg^−1^, i.p.) and perfused transcardially with 20 mL of phosphate-buffered saline (PBS), followed by 20 mL 4% paraformaldehyde (PFA) (G1101, Servicebio, China). The brains were carefully extracted from the skull and postfixed in 4% PFA for 6 h and then dehydrated with 30% sucrose at 4°C until the brain tissue sank to the bottom of the solution. Then 30-μm-thick coronal sections were prepared by a frozen microtome (CM 1950; Leica Microsystems, Germany). Free-floating sections were washed three times with PBS for 10 min each and blocked in PBS with 1% bovine serum albumin (V900933, Sigma–Aldrich, United States of America) and 0.2% Triton X-100 (T109027, Aladdin, China) for 45 min. Sections were incubated with primary antibody diluted in PBS and 0.2% Triton X-100 at 4°C in a shaker overnight. The primary antibodies used were rabbit anti-immediate early gene expression of proteins (c-Fos) (1:1,000; Cell Signaling Technology, United States). Incubated sections were washed thrice in PBS for 10 min and incubated for 2 h with a secondary antibody in PBS. The secondary antibodies used were Alexa Fluor 594 donkey anti-rabbit (1:400; Invitrogen, United States) or Alexa Fluor 488 donkey anti-rabbit (1:400; Invitrogen, United States). The samples were subsequently washed four times with PBS for 10 min each (all at room temperature). Confocal images were acquired using an automated slide scanner system (VS. 120, Olympus, Japan) and further observed and counted using OlyVIA 3.2.1 software (Olympus).

### Statistical analysis

Statistical analyses were conducted with GraphPad Prism 9.0 software (GraphPad Software). All data were presented as mean ± SEM. Unpaired *t*-tests were performed to compare the differences between the two groups, and *Welch’s* correction was used when the variance was not equal. Two-way *ANOVA* was followed by Bonferroni, Tukey, or Šídák post-tests calculate *p* values (treatment with different virus as the between-subject factors and different drugs as the within-subjects factor). Detailed descriptions can be found in the figure legends. Statistical significance was defined as *p* < 0.05.

## Results

### MS glutamatergic neurons are hyperactivated in CCI mice

**FIGURE 1:**
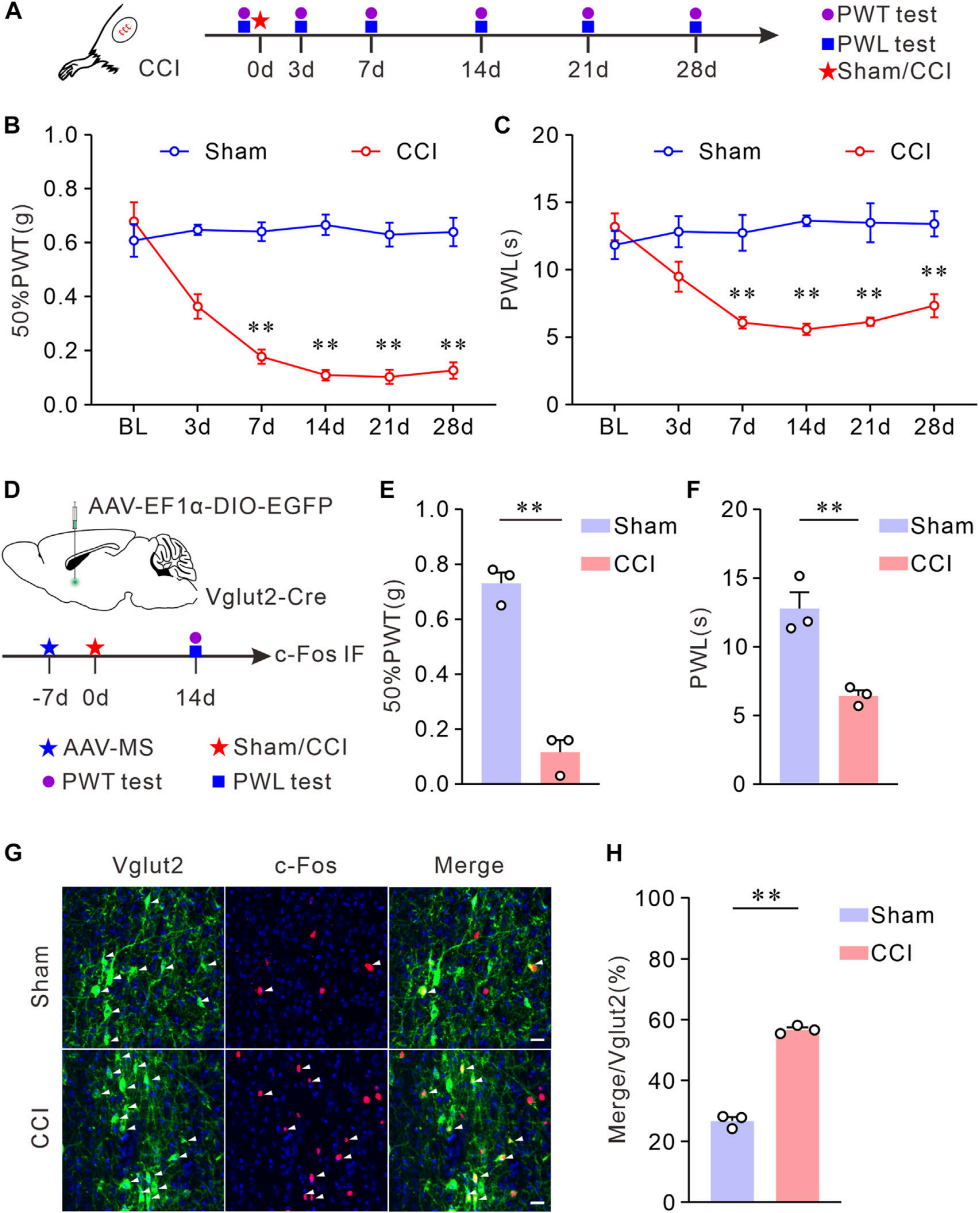
Vglut2 neurons in MS are hyperactivated on chronic neuropathic pain induced by CCI. **(A)** Schematic of CCI surgery and experimental timeline. PWTs and PWLs of the hind paws in mice were tested at day 0 before, and at days 3, 7, 14, 21, and 28 after the sham or CCI surgery. **(B,C)** The quantitative comparison of 50% PWTs **(B)** and PWLs **(C)** between the two groups. Compared with the sham mice, CCI mice exhibited a decrease in 50% PWTs and PWLs in the injured paw at days 7, 14, 21, and 28 after the CCI surgery. *n* = 8 mice/group. 50% PWT: sham *versus* CCI, 7 days *p* < 0.0001, 14 days *p* < 0.0001, 21 days *p* < 0.0001, 28 days *p* < 0.0001; PWL: sham *versus* CCI, 7 days *p* = 0.0076, 14 days *p* < 0.0001, 21 days *p* = 0.0076, 28 days *p* = 0.0018. Two-way *ANOVA* with *Bonferroni* post-tests. BL: baseline. **(D)** Experimental timeline and schematic of virus injection. Mice were injected with AAV-vectors into the MS at day 7 before the CCI surgery. 50% PWTs and PWLs were tested from day 14 after the CCI surgery. **(E,F)** Statistics demonstrated that, the CCI mice exhibited mechanical allodynia **(E)** and thermal hyperalgesia **(F)**. 50% PWT: Sham, 0.73 ± 0.04; CCI, 0.12 ± 0.04, *p* = 0.0002; PWL: Sham, 12.78 ± 1.19; CCI, 6.43 ± 0.40, *p* = 0.0036. Unpaired *t*-test: mean ± SEM. **(G)** Representative images showing Vglut2 neurons (Green) in MS of mice were co-labeled with c-Fos-positive cells (Red) at day 14 following the CCI surgery. Arrowheads indicate co-labeled neurons. Scale bar: 10 μm. **(H)** Quantification indicating that the Vglut2 neurons are hyperactivated in CCI mice. Sham: 26.71 ± 1.29, *n* = 6 slices from 3 mice; CCI: 56.70 ± 0.78, *n* = 6 slices from 3 mice, *p* < 0.0001. Unpaired *t*-test with *welch* correction. **p < 0.01. Error bars indicate SEM.

To study MS glutamatergic neuron activity in CCI mice, we measured the co-expression of c-Fos, a marker of neuronal activation (Dragunow and Faull, 1989) and Vglut2, a marker of glutamatergic neurons (Fremeau et al., 2004) in MS. First, CCI was performed to establish a neuropathic pain model. To ensure the stability of hyperalgesia in this pain model, we evaluated the pain behaviors of the injured hind paw at multiple time points following the sham or CCI surgery (Figure 1A). Compared with sham mice, CCI mice showed a long-lasting decreased 50% PWTs and PWLs (Figures 1B, C). Since both 50% PWTs and PWLs tended to be stable 14 days after the CCI surgery, we chose this time point in the experiments. Next, we labeled MS glutamatergic neurons by injecting an AAV vector (AAV-EF1α-DIO-EGFP) into MS of Vglut2-Cre mice (Figure 1D). After CCI surgery, pain behavioral tests were performed on day 14. As expected, 50% PWTs and PWLs of CCI mice declined significantly (Figures 1E, F). Then, c-Fos immunofluorescence staining was performed in MS sections of Vglut2-Cre mice to determine the neuronal activity in response to chronic neuropathic pain (Figure 1G). The staining results showed that, compared with their sham counterparts, CCI surgery induced a significant increase of c-Fos expression in MS glutamatergic neurons (Figure 1H), suggesting that these neurons were activated in CCI mice. This data indicates that the MS glutamatergic neurons are involved in chronic neuropathic pain.

### Chemogenetic activation of the Vglut2 neurons in MS induces mechanical allodynia and thermal hyperalgesia, whereas inhibition or ablation of these neurons elevates mechanical and thermal pain thresholds in naïve mice

**FIGURE 2:**
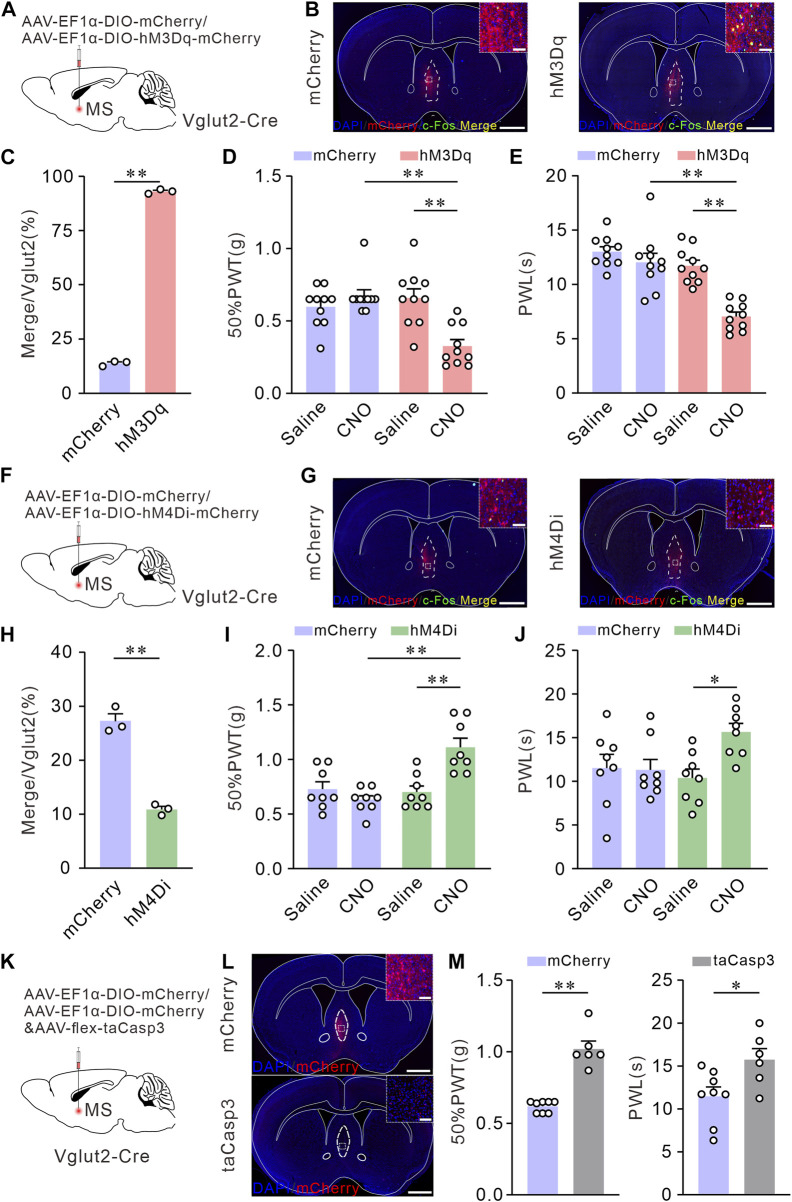
Chemogenetic activation of Vglut2 neurons in MS induces mechanical allodynia and thermal hyperalgesia, whereas inhibition or ablation of these neurons elevates mechanical and thermal pain thresholds in Naïve mice. **(A)** Schematic of virus injection. AAV-vectors were injected into the MS of Vglut2-Cre mice. **(B)** Coronal fluorescence images showing the expression of hM3Dq-mCherry in the MS Vglut2 neurons (Red). Scale bar: 1 mm. Magnified image on the top right shows the boxed area: the Vglut2 and c-Fos (Green) co-labeling neurons (Yellow) in MS in mCherry + CNO and hM3Dq + CNO groups. Scale bar: 50 μm. **(C)** Statistics showing that, the Vglut2 neurons in MS were significantly activated in hM3Dq + CNO group. *n* = 3 mice/group. Saline: 13.86 ± 0.69; CNO: 93.02 ± 0.58, *p* < 0.0001. Unpaired *t*-test: mean ± SEM. **(D,E)** Summary results showing that, compared with their control counterparts, the hM3Dq + CNO mice exhibited a decrease in 50% PWTs **(D)** and PWLs **(E)**. *n* = 10 mice/group. 50% PWT: mCherry *versus* hM3Dq, CNO, *p* < 0.0001; Saline *versus* CNO, hM3Dq, *p* = 0.0002. PWL: mCherry *versus* hM3Dq, CNO, *p* < 0.0001; Saline *versus* CNO, hM3Dq, *p* < 0.0001. Two-way *ANOVA* with *Tukey* post-tests. **(F)** Schematic of virus injection. **(G)** Coronal fluorescence images showing the expression of hM4Di-mCherry in the MS Vglut2 neurons (Red). Scale bar: 1 mm. Magnified image on the top right shows the boxed area: the Vglut2 and c-Fos (Green) co-labeling neurons (Yellow) in MS in mCherry + CNO and hM4Di + CNO groups. Scale bar: 50 μm. **(H)** Statistics showing that, the Vglut2 neurons were inhibited in hM4Di + CNO group. *n* = 3 mice/group. Saline: 27.23 ± 1.38; CNO: 10.87 ± 0.58, *p* = 0.0002. Unpaired *t*-test: mean ± SEM. **(I,J)** Summary results showing that, compared with their control counterparts, the hM4Di + CNO mice exhibited an increase in 50% PWTs **(I)** and PWLs **(J)**. *n* = 8 mice/group. 50% PWT: mCherry *versus* hM4Di, CNO, *p* < 0.0001; Saline *versus* CNO, hM4Di, *p* = 0.0005. PWL: Saline *versus* CNO, hM4Di, *p* = 0.0226. Two-way *ANOVA* with *Tukey* post-tests. **(K)** Schematic of virus injection. **(L)** Representative confocal images of genetic ablation of the Vglut2 neurons in MS (Red). Scale bar: 1 mm. Magnified image on the top right shows the boxed area. The mCherry and taCasp3 typical confocal image indicating the efficacy of taCasp3. Scale bar: 50 μm. **(M)** Summary results showing that, compared with mCherry group, the mice with taCasp3 expression exhibited an increase in 50% PWTs and PWLs. *n* = 8/6. 50% PWTs: mCherry, 0.62 ± 0.1; taCasp3, 1.02 ± 0.05, *p* < 0.0001. PWL: mCherry, 11.49 ± 1.09; taCasp3, 15.76 ± 1.27, *p* = 0.0125. Unpaired *t*-test: mean ± SEM. *p < 0.05, **p < 0.01. Error bars indicate SEM.

To further explore whether the activation of MS glutamatergic neurons leads to hyperalgesia, we applied chemogenetic stimulations to activate MS glutamatergic neurons specifically and assessed the pain thresholds in mice. AAV-EF1α-DIO-hM3Dq-mCherry (an AAV vector that expresses mCherry and hM3Dq dependence on Cre) or AAV-EF1α-DIO-mCherry (as control) was injected into the MS of Vglut2-Cre mice. 50% PWTs and PWLs were performed on day 21 (Figure 2A). Mice were injected intraperitoneally with saline (1 mg kg^-1^). Then the first determination of 50% PWTs and PWLs was considered as the baseline for the pain sensitivity. At least 1 h later, mice were subjected to an intraperitoneal injection of CNO (1 mg kg^-1^). After 30 min, 50% PWTs or PWLs were examined again. To verify the efficacy of the chemogenetics strategy, the mice were sacrificed for c-Fos immunofluorescence staining of MS sections after behavioral tests. Compared to the controls, > 90% of mCherry-positive neurons were co-labeled by c-Fos in hM3Dq group mice (Figures 2B, C), suggesting that CNO does activate these glutamatergic neurons. After CNO injection, the mice injected with AAV-EF1α-DIO-hM3Dq-mCherry exhibited a decrease in 50% PWTs and PWLs (Figures 2D, E). These data indicate that chemogenetic activation of MS glutamatergic neurons induces mechanical allodynia and thermal hyperalgesia in naïve mice.

### Chemogenetic inhibition or selective lesion of MS glutamatergic neurons elevates mechanical and thermal pain thresholds in naïve mice

We further wanted to investigate whether inhibition or lesion of MS glutamatergic neurons can improve pain thresholds. For specifical inhibition of MS glutamatergic neurons, AAV-EF1α-DIO-hM4Di-mCherry or AAV-EF1α-DIO-mCherry (as control) was injected into the MS of Vglut2-Cre mice and the pain behavioral tests were determined on day 21 (Figure 2F). The expression of chemogenetics virus was also confirmed by c-Fos immunofluorescence staining. A few Vglut2 neurons were co-labeled by c-Fos (Figures 2G, H). After CNO injection, the mice injected with hM4Di-mCherry exhibited the increased 50% PWTs and PWLs (Figures 2I, J), indicating that chemogenetic inhibition of MS glutamatergic neurons elevates mechanical and thermal pain thresholds in naïve mice.

In addition, we selectively lesioned the MS glutamatergic neurons by injecting the mixture of AAV-EF1α-DIO-mCherry and AAV-flex-taCasp3-TEVp. The controls only were injected with AAV-EF1α-DIO-mCherry (Figure 2K). We confirmed cell death by confocal fluorescent imaging. Compared to the controls, less number of mCherry neurons were presented on MS with AAV-flex-taCasp3-TEVp injection (Figure 2L). 50% PWTs and PWLs were tested on day 21. Similarly, mice injected with AAV-flex-taCasp3-TEVp exhibited an increase in 50% PWTs and PWLs (Figure 2M), demonstrating that selective lesion of MS glutamatergic neurons improves mechanical and thermal pain thresholds in naïve mice.

### Chemogenetic inhibition of MS glutamatergic neurons relieves chronic neuropathic pain induced by CCI

**FIGURE 3:**
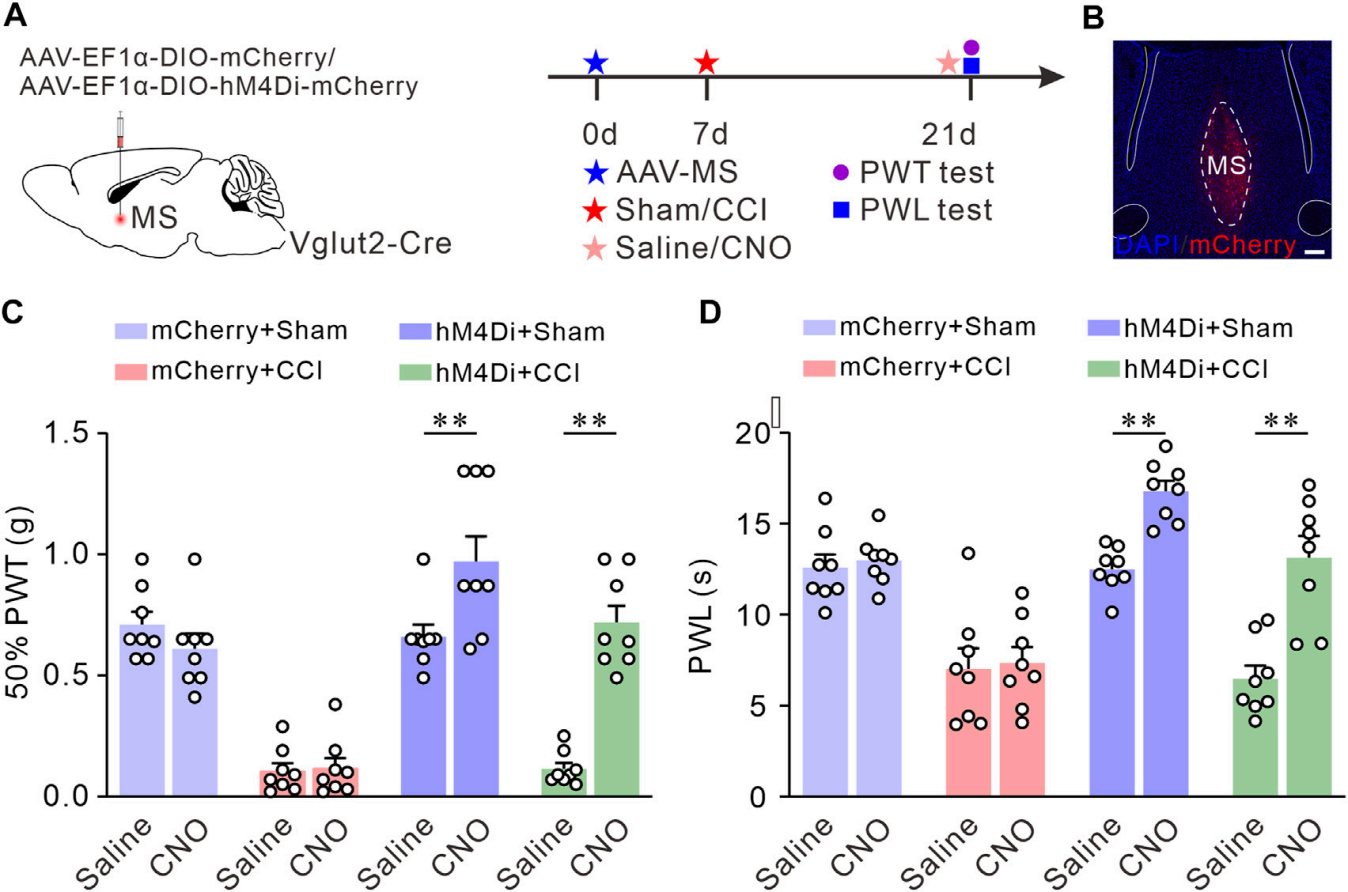
Chemogenetic inhibition of the Vglut2 neurons in MS relieves chronic neuropathic pain induced by CCI. **(A)** The diagram of virus injection and experimental timeline. **(B)** Example confocal images of hM4Di-mCherry expression in the MS Vglut2 neurons (Red). **(C,D)** Summary results showing that, compared with their control counterparts, the hM4Di + CCI + CNO mice exhibited an increase in 50% PWTs **(C)** and PWLs **(D)**. *n* = 8 mice/group. 50% PWT: Saline *versus* CNO, hM4Di + CCI, *p* < 0.0001. PWL: Saline *versus* CNO, hM4Di + CCI, *p* < 0.0001. Two-way *ANOVA* with *Bonferroni* post-tests. **p < 0.01. Error bars indicate SEM.

Next, we investigated the pain-relieving effect of MS glutamatergic neurons in the CCI-induced neuropathic pain model mice. We targeted MS glutamatergic neurons by injecting AAV-EF1α-DIO-hM4Di-mCherry or AAV-EF1α-DIO-mCherry (as control) into the MS of Vglut2-Cre mice. Subsequent sham or CCI surgery was performed, respectively. 50% PWTs and PWLs were measured 14 days after surgery (Figure 3A). MS sections were prepared to verify the expression of hM4Di-mCherry (Figure 3B). The results showed that the pain thresholds in CCI mice injected with AAV-EF1α-DIO-hM4Di-mCherry and CNO did not decline as much as those of CCI control mice (Figures 3C, D). These findings suggest that chemogenetic inhibition of the MS glutamatergic neurons relieves chronic neuropathic pain induced by CCI.

### LH and SuM receive direct projections from MS glutamatergic neurons

**FIGURE 4:**
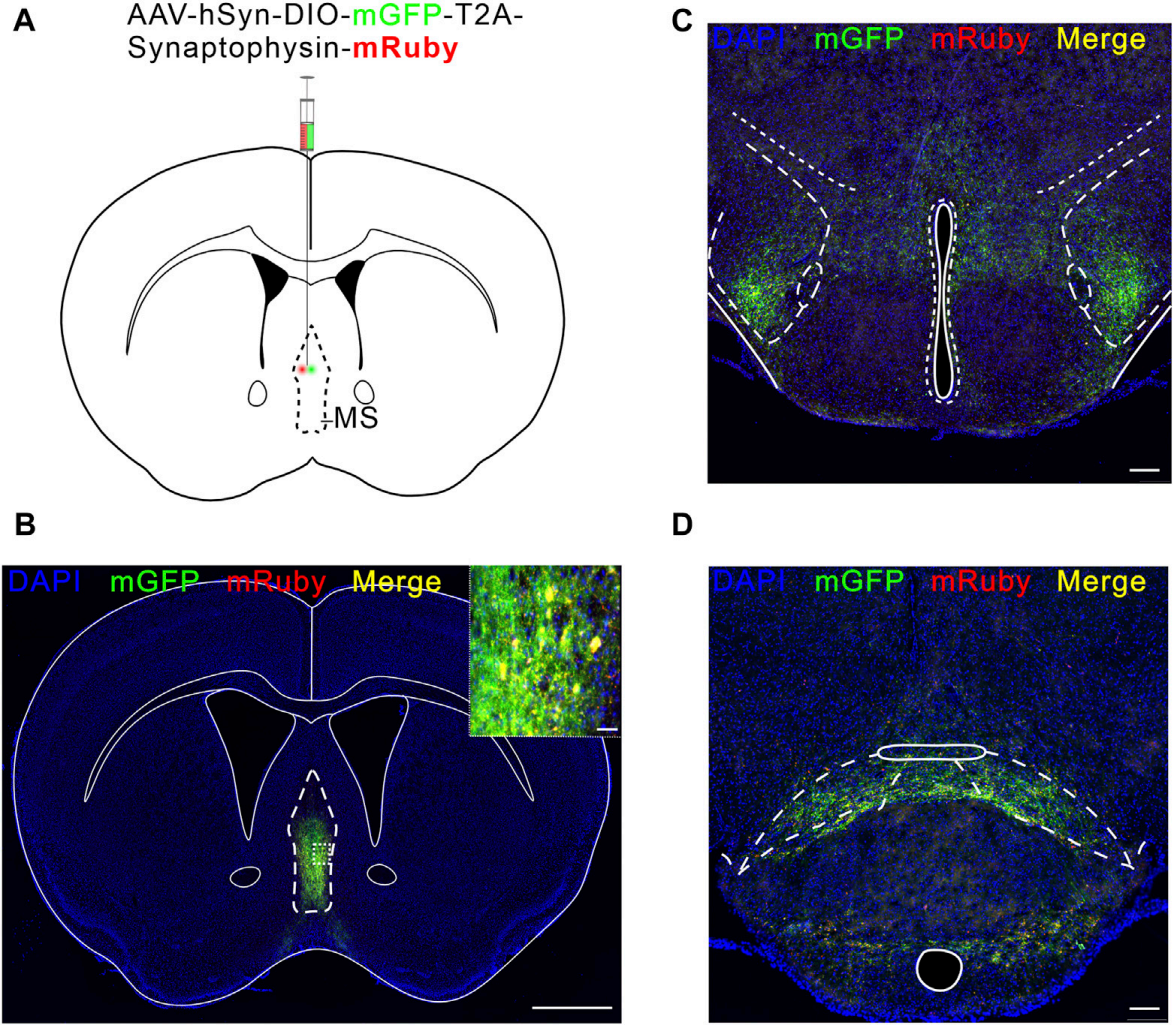
LH and SuM receive direct projections from MS Vglut2 neurons. **(A)** Schematic of AAV-hSyn-DIO-mGFP-T2A-Synaptophysin-mRuby injection into MS of Vglut2-Cre mice. **(B)** Fluorescence image of mGFP-mRuby expression in MS. Scale bar: 1 mm. The magnified image on the top right shows the expression of mGFP-mRuby (Green-Red) *in situ*. Scale bar: 50 μm. **(C,D)** Example confocal images of mGFP-mRuby expression in LH **(C)** and SuM **(D)**. Scale bar: 200 μm.

We next investigated how MS glutamatergic neurons are involved in pain regulation. In this part, we explored the MS glutamatergic downstream pathways mediating neuropathic pain. We used an anterograde, viral tracing method (Knowland et al., 2017) to examine the brain regions receiving direct projections from MS glutamatergic neurons. AAV-hSyn-DIO-mGFP-T2A-Synaptophysin-mRuby was injected into the MS of Vglut2-Cre mice (Figures 4A, B). Synaptophysin-mRuby is a fusion protein of mRuby and Synaptophysin. Synaptophysin proteins are located on synaptic vesicles and are used to mark the location of synapses (Tsetsenis et al., 2021). Although mGFP-positive fibers are present in some brain regions, this does not mean MS glutamatergic neurons form synapses in these nuclei but only cross these pathways. Therefore, the co-existence of mGFP and synaptophysin-mRuby in LH and SuM suggests a direct projection relationship between MS and these two nuclei (Figures 4C, D).

### MS Vglut2-LH projections bilaterally regulate mechanical and thermal pain thresholds in naïve mice

**FIGURE 5:**
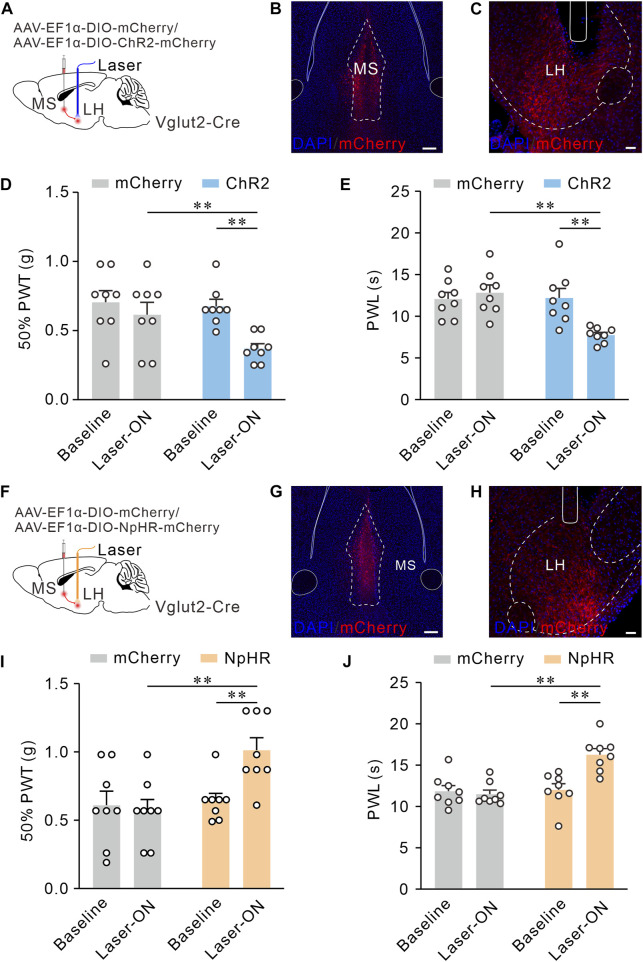
MS Vglut2-LH projections bilaterally regulate mechanical and thermal pain thresholds in naïve mice. **(A,F)** Schematic drawing of virus injection. **(B,G)** Typical confocal images of ChR2-mCherry and NpHR-mCherry expression in MS Vglut2 neurons, respectively. Scale bar: 200 μm. **(C,H)** Sample confocal images showing the terminals of MS Vglut2 neurons in LH and the tips of optical fibers above LH. Scale bar: 50 μm. **(D,E)** Statistics showing that, the ChR2 mice exhibited a decrease in 50% PWTs **(D)** and PWLs **(E)** by optogenetic activation of MS Vglut2-LH projections, *n* = 8 mice/group. 50% PWT: Baseline *versus* Laser-ON, ChR2, *p* = 0.0005. PWL: Baseline *versus* Laser-ON, ChR2, *p* = 0.0006. **(I,J)** Statistics showing that, optogenetic inhibition of MS Vglut2-LH projections increase mechanical **(I)** and thermal pain thresholds **(J)** in naïve mice. *n* = 8 mice/group. 50% PWT: Baseline *versus* Laser-ON, NpHR, *p* = 0.0114. PWL: Baseline *versus* Laser-ON, NpHR, *p* < 0.0001. Two-way *ANOVA* with Šídák post-tests. **p < 0.01. Error bars indicate SEM.

We have determined the MS glutamatergic neurons mainly project to LH and SuM. Next, we planned to confirm the functional role of these projections in mediating pain sensitivity. Here, through optogenetics, we observe the real-time effects of MS Vglut2-LH projections intervention on pain behaviors. For optogenetic activation of MS Vglut2-LH projections, AAV-EF1α-DIO-hChR2 (H134R)-mCherry or AAV-EF1α-DIO-mCherry (as controls) was injected into MS, and optical fibers were implanted above LH of Vglut2-Cre mice (Figures 5A–C). According to the previous study (An et al., 2021), to activate MS glutamatergic neurons, we randomly applied 10 Hz blue laser stimulations. 50% PWTs and PWLs were examined when the mice have subjected to the blue laser (470 nm) stimulation by optical fiber coupled with a laser generator. The data showed that the mice injected with hChR2 (H134R)-mCherry and received laser stimulation had lower 50% PWTs and PWLs than the controls (Figures 5D, E), suggesting that optogenetic activation of MS glutamatergic neurons promotes pain sensitivity. For optogenetic inhibition of MS Vglut2-LH projections, AAV-EF1α-DIO-NpHR3.0-mCherry or AAV-EF1α-DIO-mCherry (as controls) was injected into MS, and optical fibers were implanted above LH of Vglut2-Cre mice (Figures 5F–H). After 21 days, yellow light (589 nm) was delivered to LH by optical fiber connected to a laser generator. The result showed that, compared to the controls, mice injected with NpHR3.0-mCherry and received laser stimulation had an increase in 50% PWTs and PWLs (Figures 5I, J). Together, we demonstrated that MS Vglut2-LH projections bilaterally regulate pain sensitivity.

### MS Vglut2-SuM projections have no effect on pain thresholds in naïve mice

**FIGURE 6:**
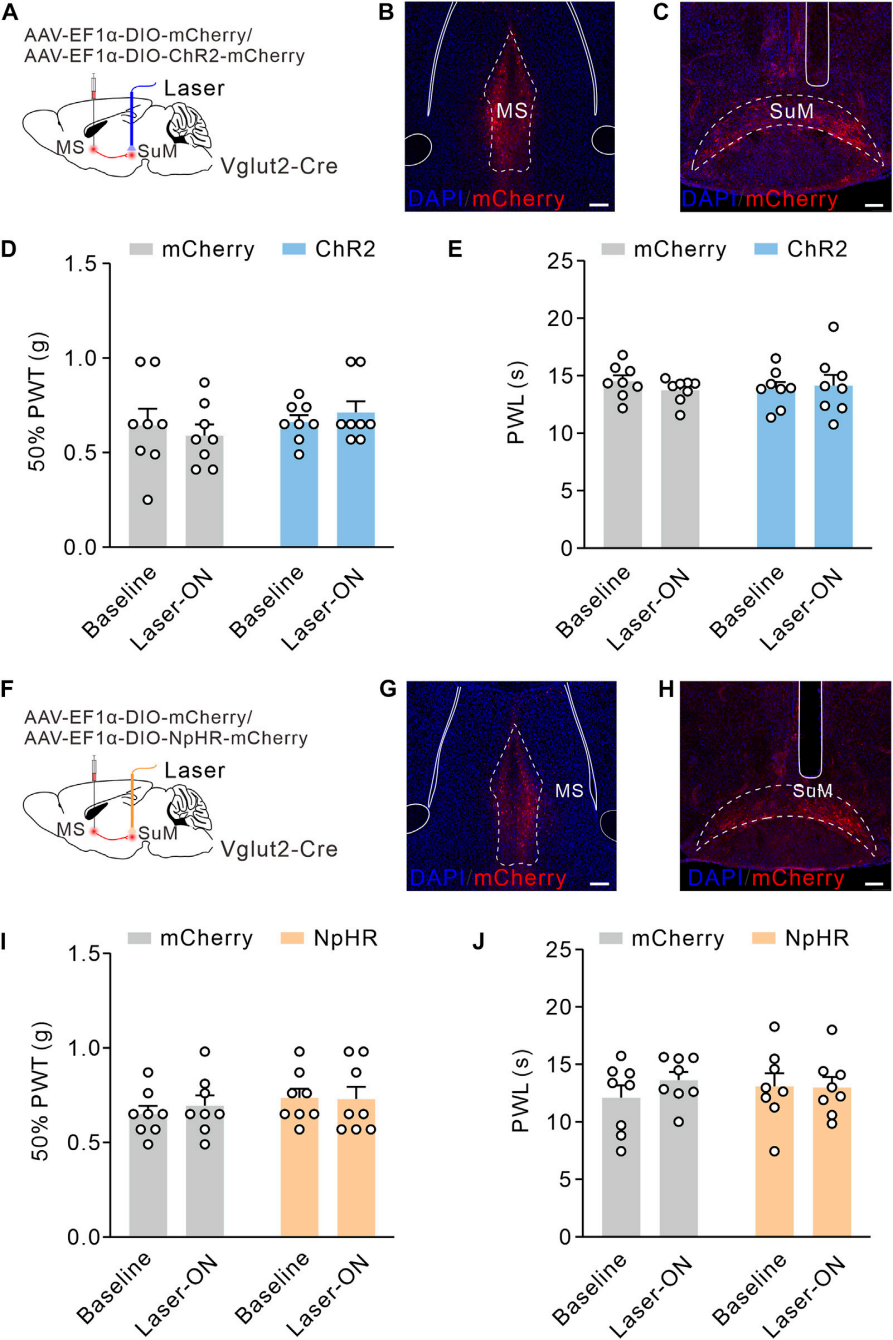
MS Vglut2-SuM projections have no effect on pain thresholds in naïve mice. **(A,F)** Schematic drawing of virus injection. **(B,G)** Typical confocal images of ChR2-mCherry and NpHR-mCherry expression in MS Vglut2 neurons, respectively. Scale bar: 200 μm. **(C,H)** Sample confocal images showing the terminals of MS Vglut2 neurons in SuM and the tips of optical fibers above SuM. Scale bar: 200 μm. **(D,E,I,J)** Statistics showing that, neither optogenetics activation **(D,E)** nor inhibition **(I,J)** of the MS Vglut2-SuM projections caused statical differences for 50% PWTs and PWLs in naïve mice, compared to their counterparts. Two-way ANOVA with Šídák post-tests. Error bars indicate SEM.

According to the same procedures, we activated MS Vglut2-SuM projections by optogenetics (Figures 6A–C). Surprisingly, we found no significant difference between baseline and laser-on groups in the 50% PWTs and PWLs (Figures 6D, E). Similarly, optogenetic inhibition of MS Vglut2-SuM projections (Figures 6F–H) did not influence 50% PWTs or PWLs (Figures 6I, J). Hence, revealing that MS Vglut2-LH, but not MS Vglut2-SuM projections regulate mechanical and thermal pain thresholds in naïve mice.

### Optogenetic inhibition of MS Vglut2-LH projections relieves chronic neuropathic pain induced by CCI

**FIGURE 7:**
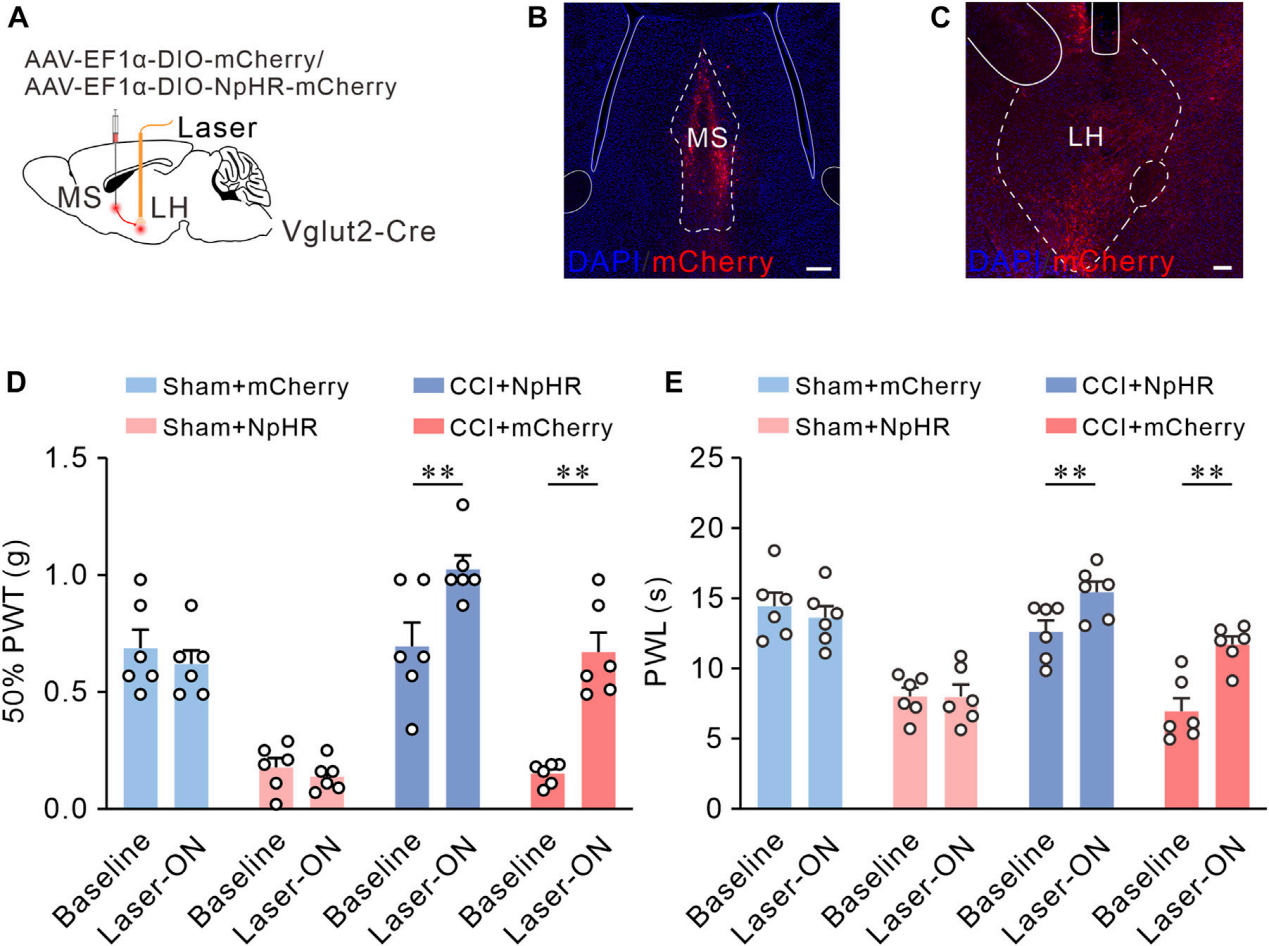
Optogenetic inhibition of MS Vglut2-LH projections relieves chronic neuropathic pain induced by CCI. **(A)** The diagram of virus injection. **(B)** Confocal image showing ChR2-mCherry expression in MS Vglut2 neurons. Scale bar: 200 μm. **(C)** Confocal images showing the terminals of MS Vglut2 neurons in LH and the tips of optical fibers above LH. Scale bar: 200 μm. **(D,E)** Summary results showing that, the NpHR + Laser-ON mice exhibited increased 50% PWTs and PWLs, compared with their control counterparts. *n* = 6 mice/group. 50% PWT: Baseline *versus* Laser-ON, NpHR + CCI, *p* < 0.0001. PWL: Baseline *versus* Laser-ON, NpHR + CCI, *p* < 0.0001. Two-way *ANOVA* with Šídák post-tests. **p < 0.01. Error bars indicate SEM.

Since inhibition of MS Vglut2-LH projections elevated pain thresholds in naïve mice, we inferred that these projections also relieve chronic neuropathic pain induced by CCI. To test this hypothesis, we utilized optogenetic tools to inhibit MS Vglut2-LH projections in CCI mice. The mice were injected with AAV-EF1α-DIO-NpHR3.0-mCherry or AAV-EF1α-DIO-mCherry (as controls) 7 days before sham or CCI surgery. Twenty-one days after the AAV injection, 50% PWTs, and PWLs were checked (Figures 7A–C). Statistics showed that there were no differences in mCherry group mice. Compared to the laser-off mice, 50% PWTs and PWL increased in the NpHR3.0-mCherry mice with laser-on (Figures 7D, E). The results suggested that inhibition of MS Vglut2-LH projections relieves chronic neuropathic pain induced by CCI.

## Discussion

Evidence from rodent studies suggests that MS is implicated in processing and regulation pain (Ang et al., 2017). Studies have shown that MS neurons can be activated by both acute or chronic nociceptive stimulation (Dutar et al., 1985; Burstein et al., 1987; Jiang et al., 2018a). The results of pharmacological tests demonstrated that inhibition of the MS reduced experimental neuropathic pain in mice and inhibited formalin-induced licking and flinching in rats (Lee et al., 2011; Ariffin et al., 2018). Besides, the analgesic effects of general anesthesia are prolonged by lesion or inactivation of MS (Ma et al., 2002; Leung et al., 2013). These results prove that MS is essential in the regulation of nociception. However, how the individual neuronal populations of MS regulate pain perception remains a questionable phenomenon. The MS consists of three major neuronal populations: cholinergic (about 47%), glutamatergic (about 25%), and GABAergic neurons (about 28%) (Griffith & Matthews, 1986; Markram & Segal, 1990; Dutar et al., 1995; Colom et al., 2005; Yu et al., 2022). Previous findings (Jiang et al., 2018a; Jiang, et al., 2018b) suggest that cholinergic neurons in MS are involved in encoding hyperalgesia and anxiety-like behaviors in mice with chronic pain induced by the complete Freund’s adjuvant (CFA). Intraplantar injection of CFA increased the number of c-Fos-positive neurons in MS cholinergic neurons (Jiang et al., 2018a). Chemogenetic inhibition of MS cholinergic neurons attenuated perceptual (Jiang, et al., 2018b) and anxiety-like (Jiang et al., 2018a) behaviors of chronic pain induced by CFA injection in mice. Interestingly, another study (Ang et al., 2015) found that selective lesions of MS GABAergic neurons did not affect the abnormal pain-perceptual behavior but attenuated the conditioned place aversion induced by formalin injection. However, we found that chronic neuropathic pain caused by CCI activates MS glutamatergic neurons, and inhibition of MS glutamatergic neurons reverses nociceptive sensitization induced by CCI. Our results demonstrate that MS glutamatergic neurons play a critical role in perceiving and regulating nociceptive sensitization in chronic neuropathic pain.

MS is a part of the pain system in the brain (Takeuchi et al., 2021). On the one hand, the MS receives afferents from the nociceptive system, and peripheral nociceptive stimuli activate MS neurons (Dutar et al., 1985; Burstein et al., 1987; Jiang, et al., 2018b) and receives neural afferents from a cluster of nuclei that have been shown to be involved in pain regulation (Ang et al., 2017), including LH (Fakhoury et al., 2020), the lateral septal nucleus (LS) (Wang et al., 2023), and the ventral tegmental area (VTA) (Zhang et al., 2017). On the other hand, the MS receives afferents from the nociceptive system, and peripheral nociceptive stimuli activate MS neurons. MS regulates pain by directly or indirectly innervating regions of the nociceptive system (Ang et al., 2017). Neurons in MS are known to send neural efferences to nuclei involved in sensory information transmission, including the anterior cingulate cortex (ACC), raphe nucleus (RN), LH, and SuM (Takeuchi et al., 2021). Furthermore, inhibition of the MS-rACC cholinergic pathway suppresses rACC pyramidal neuronal activities and relieves CFA-induced inflammatory pain (Jiang, et al., 2018b). Interestingly, in anesthetized animals, electrical stimulation of the MS inhibited the firing rate of wide dynamic range neurons in the spinal cord dorsal horn evoked by the peripheral noxious stimuli (Carstens et al., 1982; Hagains et al., 2011). This implies that MS might modulate pain by acting on the descending pain inhibitory system. Indeed, the evidence above suggests that MS may be a critical hub for the reception and processing of nociceptive stimulus signals. The current study demonstrates that chronic neuropathic pain increases the activity of MS glutamatergic neurons and MS glutamatergic neural population modulates the hyperalgesia of chronic neuropathic pain via their projections to LH.

LH is a heterogeneous brain region composed mainly of orexinergic, glutamatergic, GABAergic, and various neuropeptide-expressing neurons (Fakhoury et al., 2020).The LH regulates many processes, including feeding, sleep, arousal, and pain (Fakhoury et al., 2020; An et al., 2021; Xiang et al., 2022; Wang et al., 2023) and electrical stimulation of the LH region induces analgesic effects (Fakhoury et al., 2020). It has been confirmed that orexinergic neurons and parvalbumin (PV)-positive neurons of the LH play an essential role in the process of pain regulation (Inutsuka et al., 2016; Siemian et al., 2021). Specifically, activation of LH orexinergic or PV-positive neuronal population attenuated formalin-induced pain-related behaviors in mice. However, little is known about the role of other types of neurons in the LH in pain regulation. Interestingly, an *in vivo* study showed that optogenetic inhibition of MS-LH pathway neurons attenuated the firing frequency of LH glutamatergic neurons in wake-state mice (An et al., 2021). Therefore, we hypothesized that the MS-LH glutamatergic pathway might regulate pain by acting on LH glutamatergic neurons. Although it has been confirmed that the activation of LH glutamatergic neurons can cause aversion, arousal, promote-recovery from general anesthesia, escape behavior, and defensive behavior (Nieh et al., 2016; Li et al., 2018; de Jong et al., 2019; Chen et al., 2020; Wang et al., 2021b; Zhao et al., 2021). But there is still a lack of reliable evidence to confirm the regulatory role of LH glutamatergic neurons on pain, which may become a direction for future research. Furthermore, the synaptic terminals of orexinergic neurons exhibit co-localization with Vglut2 markers, indicating they are capable of rapid, synaptic glutamate release (Rosin et al., 2003; Henny et al., 2010; Bonnavion et al., 2016). Therefore, further confirmation is required to determine whether the MS-LH glutamatergic pathway targets LH orexinergic neurons.

Previous studies have found that the SuM plays a role in modulating arousal, locomotion, and feeding processes (Pedersen et al., 2017; Farrell et al., 2021; Chen et al., 2022). Besides, studies also indicate that the SuM is involved in the processing of nociceptive information (Borhegyi & Freund, 1998; Ma & Leung, 2006; Ariffin et al., 2010; Ang et al., 2017). Microinjection of nicotinic antagonists into the SuM selectively attenuated the hippocampal responses elicited by formalin-induced nociceptive stimuli (Ariffin et al., 2010), and inactivation of the SuM prolongs the duration of antinociceptive function of general anesthetics (Ma & Leung, 2006). The present study shows that MS glutamatergic neurons export numerous neuronal terminals to the SuM. Nevertheless, our results found that activation or inactivation of the MS-SuM glutamatergic pathway did not alter responses to noxious stimulation in naive mice. However, activation of the MS-LH glutamatergic pathway enhanced the sensitivity to nociceptive stimulation in naive mice. In contrast, inhibition of the MS-LH glutamatergic pathway attenuated the sensitivity to nociceptive stimulation in naive mice and reversed the hyperalgesia in CCI mice. Our results confirms that the modulation of nociception by MS glutamatergic neurons depends on their specific downstream projection target region.

Although, few researchers have made progress in studying the circuit mechanism of chronic neuropathic pain (Zhang et al., 2017; Wang et al., 2021c). But the neural circuit mechanism of MS regulating chronic neuropathic pain has been rarely reported. In the present study, we have identified an important role of MS glutamatergic neurons in regulating chronic neuropathic pain, and these MS glutamatergic neurons regulate nociception through the MS-LH pathway but not the MS-SUM pathway. Our study identifies novel cellular and neural circuit mechanisms that regulate chronic neuropathic pain, which may provide valuable therapeutic targets for chronic pain.

## Data Availability

The original contributions presented in the study are included in the article/supplementary material, further inquiries can be directed to the corresponding authors.
